# Supplementary material for: Quantitative assessment can stabilize indirect reciprocity under imperfect information
Source: Nat Commun. 2023 Apr 12;14:2086. doi: 10.1038/s41467-023-37817-x (PMC10097696; doi:10.1038/s41467-023-37817-x)
Supplement: Supplementary file 1 — Supplementary Information [file 41467_2023_37817_MOESM1_ESM.pdf]

Quantitative assessment can stabilize indirect reciprocity under imperfect information

Laura Schmid[1,*], Farbod Ekbatani[2], Christian Hilbe[3], Krishnendu Chatterjee[1]
[1]KAIST Graduate School of AI, 02455 Seoul, South Korea
[2]Booth School of Business, The University of Chicago, Chicago, Illinois 60637, USA
[3]Max Planck Research Group Dynamics of Social Behavior, Max Planck Institute for Evolutionary Biology, 24306 Ploen, Germany
[*]E-mail: laura.schmid@kaist.ac.kr

March 16, 2023

In the following, we present theoretical underpinnings of our main results, thus further strengthening our findings. In **Supplementary Note 1**, we analyze reputation dynamics in a homogeneous population of leading eight players using quantitative assessment that start out with a single disagreement among them. We model the dynamics as a Markov chain, and show that the expected time to recovery from a single disagreement is bounded from above by the corresponding quantity in the binary assessment model.

In **Supplementary Note 2**, we give a formal characterization of those social norms that are successful by using quantitative assessment under private and imperfect information. This is in analogy to previous work that gave such axioms for the case of binary assessment under public information[2]. With this characterization, we can explain why we have identified four leading eight norms that can maintain cooperation in the private information setting when players use more nuanced assessment.

Supplementary Note 1: Recovery analysis

We can also analyze the recovery from single disagreements when $R = 1$, in close analogy to previous work[1]. To this end, we consider a setting where observation is perfect ($q = 1$) and perception errors are rare ($\varepsilon \to 0$). We then assume an initial configuration where all players perceive everyone else as good with the exception of player 1, who perceives player 2 as bad, potentially due to a previous error. That is, we have an initial image matrix $M(0)$ with entries

$$r_{ij}^0 = \begin{cases} -1 & \text{if } i = 1, j = 2 \\ 0 & \text{otherwise.} \end{cases} \tag{1}$$

The defining feature of this initial configuration is that with the exception of the pair $(i, j) = (1, 2)$, all players assign the overall label ”good” to their co-players. Alternatively, we can thus also envision an intial configuration of

$$r_{ij}^0 = \begin{cases} -1 & \text{if } i = 1, j = 2 \\ 1 & \text{otherwise.} \end{cases} \tag{2}$$

The defining feature is that with the exception of the pair $(i, j) = (1, 2)$, all players assign the overall label "good" (henceforth denoted G) to their co-players.

We define as recovery of the population the return to the state where all players have a good reputation, starting from $M(0)$. In this context, we are now interested in two quantities, which depend on the social norm L_i applied in the population: the population's recovery probability ρ_i, and the expected time till recovery τ_i, conditioned on recovery actually taking place.

With the following proposition, we simplify our analysis:

Proposition 1. *Consider the indirect reciprocity game for a population in which everyone applies the same leading-eight strategy L_i and uses quantitative assessment with $R = 1$, such that reputation scores can take the values $r_{ij} = \{-1, 0, 1\}$. Moreover, assume that the initial image matrix is $M(0)$ as defined either by Eq.(1) or (2), and let $M(t)$ denote the image matrix at some subsequent time $t > 0$ according to the process with perfect observation and no noise, $q = 1$ and $\varepsilon = 0$. Then, $M(t) \in \mathcal{M}$, where $\mathcal{M}$ is the set of all image matrices that satisfy the following four conditions*

$$\text{(i) } r_{ii} \in \{0, 1\} \text{ for all } i, \quad \text{(ii) } r_{ij} = r_{i'j} \text{ for } i, i' \geq 2, j \geq 1, \quad \text{(iii) } r_{ij} \in \{0, 1\} \text{ for all } \{i, j\} \geq 2, \tag{3}$$

Additionally, we have that

$$\text{(iv) } r_{ij}^{t+1} \geq r_{ij}^t \text{ for all } i, j \geq 2, \tag{4}$$

and that

$$\text{(v) } r_{ii}^{t+1} \geq r_{ii}^t \text{ for all } i. \tag{5}$$

Proof of Proposition 1. We consider the Markov chain on the space of image matrices $H = (h_{M,M'})$, in the limiting case of $\varepsilon = 0$ and $q = 1$. We then show that the set $\mathcal{M}$ of image matrices that satisfy the properties (i)–(v) is invariant. That is, let $M \in \mathcal{M}$ be arbitrary and suppose that $h_{M,M'} > 0$ for some matrix M'. Then also $M' = \{r'_{ij}\}$ satisfies all properties.

(i) $r'_{ii} \in \{0, 1\}$ for all i. Since $M \in \mathcal{M}$, initially all players consider themselves as good, i.e. $r_{ii}^0 \in \{0, 1\}$. All leading-eight strategies have the property that the strategy's action rule prescribes an action that lets a good donor maintain her good reputation in her own eyes, independent of which reputation she assigns to the recipient. Thus, all players keep considering themselves as good after one interaction; either they do not need to make a decision (because they were not chosen to act as the donor), or they choose an action they themselves evaluate as good.

(ii)– *(iii)* $r'_{ij} = r_{i'j}$ for $i, i' \geq 2, j \geq 1$ and $r_{ij} \in \{0, 1\}$ for all $i, j \geq 2$. Since $M \in \mathcal{M}$, all players $i, j \geq 2$ initially agree on the reputations of all population members. Because they all apply the same

assessment rule and observation errors are excluded, they also agree on how the donor's action in the subsequent interaction needs to be assessed. This shows $r'_{il} = r'_{jl}$ for all $i, j \geq 1$ and all l. Moreover, since all players $i, j \geq 2$ consider each other as good initially, and since their common action rule only lets them choose actions that let them keep their good reputation, we conclude $r'_{ij} \in \{0, 1\}$ for $i, j \geq 2$.

(*iv*)– (*v*) $r_{ij}^{t+1} \geq r_{ij}^{t}$ for all $i, j \geq 2$ and $r_{ii}^{t+1} \geq r_{ii}^{t}$ for all i. Since $M \in \mathcal{M}$, all players $i \geq 2$ initially agree on the good reputations of all population members. By (ii) and (iii), all players $i \geq 2$ also keep their good image of each other, and only potentially change their opinion about player 1. Since they thus never act against their assessment of each other for any reason, their reputation scores in each others' eyes can only increase. Hence $r'_{ij} \geq r_{ij}$ for all $i, j \geq 2$. The same reasoning applies to all self images, including that of player 1 - due to the lack of observation errors of any kind, players never act against their own assessment rule, and can only improve their self image over time. This shows $r'_{ii} \geq r_{ii}$ for all i.

□

Proposition 1 guarantees that when we consider a process with private information, perfect observation, and no noise, (i) all players assign themselves a good reputation overall, (ii) all players $2 \leq i, j \leq N$ assign each other a good reputation overall, (iii) all players $2 \leq i, j \leq N$ assign the same reputation to player 1, (iv) the exact reputation scores that players $2 \leq i, j \leq N$ assign to each other at time t can not be smaller than in the initial configuration. Furthermore, (v) and (vi) additionally imply that the exact reputation scores that players $2 \leq i, j \leq N$ assign to each other are nondecreasing over all timesteps t, which means that the overall reputations among these players cannot turn into "bad".

Proposition 1 also lets us reduce the state space when we consider a model of private information. Instead of tracking entire image matrices M, we can focus on 3-tuples (s, k, l), with $s \in \{-1, 0, 1\}$, $k \in \{0, ..., N-1\}$, $l \in \{0, ..., N-1\}$ and $0 \leq (k+l) \leq N-1$. We identify s as player 1's reputation score from the perspective of all other players (due to Proposition 1(iii), all other players agree on player 1's reputation). The value of k denotes the number of players that player 1 considers to have score $r_{1i} = 0$, whereas l denotes the number of players that 1 considers to have score $r_{1j} = 1$. The sum of k and l thus corresponds to the number of players that player 1 considers to have a good reputation overall. We can use this reduction due to Proposition 1(iii), which says that all other players can be considered to be equivalent.

In this reduced state space, the Markov chain has $\frac{3(N+1)N}{2}$ states in total. The initial states as defined in Eqs.(1) and (2) now correspond to the 3-tuples $(0, N-2, 0)$ and $(1, 0, N-2)$. On the other hand, we can identify the fully recovered state as a group of configurations $\mathcal{A}$, with

$$\mathcal{A} = \{(x, y, z) | x \in \{0, 1\}, y + z = N - 1\} \tag{6}$$

We can now write down transition probabilities for L_i, $f^i(s, k, l; s', k', l')$, for the reduced state

space. They denote probabilities of the population moving from state (s,k,l) to (s',k',l') in one round. There are at most 12 different transitions the population can take in the course of a run of the reputation dynamics. Given the nature of the quantitative assessment dynamics we have introduced, many transition probabilities are independent of the exact value of s; instead, they depend on whether $s \geq S$ with S the threshold for overall assessment, i.e. whether player 1 has a "good" or "bad" image in the eyes of the other players. This means that in these cases $f^i(1,k,l;1,k',l') = f^i(0,k,l;0,k',l')\ \forall i,k,l$, and we write them as $f^i(G,k,l;G,k',l')$. In analogy, we write $f^i(B,k,l;B,k',l')$ for $f^i(-1,k,l;-1,k',l')$.

We can calculate the transition probabilities as follows:

Transition $(G,k,l) \to (G,k{+}1,l)$. This case can only occur if a player $i > 1$ is chosen to be the donor who is perceived as bad by player 1. Given that the current state is (G,k,l), it follows from Proposition 1 that the donor considers everyone as good, and hence they cooperate. If player 1 considers the receiver to be good, this leads them to assign a good reputation to the donor, independent of the applied leading-eight strategy L_i. Otherwise, if player 1 considers the receiver to be bad, the donor only obtains a good reputation for L_1, L_2, L_3, and L_5. Therefore, the corresponding transition probability is

$$f^i(G,k,l;\ G,k{+}1,l) = \begin{cases} \frac{N-(k+l)-1}{N} & \text{if } i \in \{1,2,3,5\} \\ \frac{N-(k+l)-1}{N}\frac{k+l+1}{N-1} & \text{if } i \in \{4,6,7,8\}. \end{cases} \tag{7}$$

Transition $(G,k,l) \to (G,k{-}1,l)$. This case can only occur if a player $i > 1$ is randomly chosen to act as the donor who is perceived to have reputation score $r_{1i} = 0$ by player 1. Similar to before, player i will always cooperate, which is only considered as bad by player 1 if the receiver is considered as bad by player 1 and if the applied strategy is either L_2, L_5, L_6, or L_8. Therefore, the transition probability is

$$f^i(G,k,l;\ G,k{-}1,l) = \begin{cases} 0 & \text{if } i \in \{1,3,4,7\} \\ \frac{k}{N}\frac{N-(k+l)-1}{N-1} & \text{if } i \in \{2,5,6,8\}. \end{cases} \tag{8}$$

Transition $(G,k,l) \to (B,k,l)$. This corresponds to the probability $f^i(0,k,l;-1,k,l)$. The transition can only occur if player 1 is chosen to be the donor, and if player 1 defects against the receiver (which in turn requires player 1 to consider the receiver as bad). The corresponding transition probability is

$$f^i(0,k,l;\ -1,k,l) = \frac{1}{N}\frac{N-(k+l)-1}{N-1}. \tag{9}$$

Transition $(B,k,l) \to (B,k{+}1,l)$. This case requires that a player $i > 1$ is chosen to be the donor who is considered as bad by player 1. This donor cooperates, unless the randomly chosen receiver happens to be player 1 (who is bad from the perspective of all other players). Thus, player 1 considers the donor as good after this round unless the receiver is player 1, or the receiver is a group member that is considered as bad by player 1 and the applied leading-eight strategy is

L_4, L_6, L_7, or L_8. Hence, we obtain

$$f^i(B,k,l;\ B,k{+}1,l) = \begin{cases} \frac{N-(k+l)-1}{N}\frac{N-2}{N-1} & \text{if } i \in \{1,2,3,5\} \\ \frac{N-(k+l)-1}{N}\frac{(k+l)}{N-1} & \text{if } i \in \{4,6,7,8\}. \end{cases} \tag{10}$$

Transition $(B,k,l) \to (B,k{-}1,l)$. This case requires that a player $i > 1$ is chosen to be the donor who player 1 considers to have reputation score $r_{1i} = 0$. To become bad in player 1's eyes, this donor then either needs to defect against player 1, or he needs to cooperate against a receiver who is considered as bad by player 1 (provided that the applied leading-eight strategy is L_2, L_5, L_6, or L_8). The transition probability becomes

$$f^i(B,k,l;\ B,k{-}1,l) = \begin{cases} \frac{k}{N}\frac{1}{N-1} & \text{if } i \in \{1,3,4,7\} \\ \frac{k}{N}\frac{N-(k+l)}{N-1} & \text{if } i \in \{2,5,6,8\}. \end{cases} \tag{11}$$

Transition $(B,k,l) \to (G,k,l)$. This corresponds to the probability $f^i(-1,k,l;0,k,l)$. It requires player 1 to be the donor, and that player 1 cooperates with her co-player. The probability is

$$f^i(-1,k,l;\ 0,k,l) = \frac{1}{N}\frac{(k+l)}{N-1}. \tag{12}$$

Transition $(G,k,l) \to (G,k-1,l+1)$. This case requires that a player $i > 1$ is chosen to be the donor who is considered to have reputation score $r_{1i} = 0$ by player 1. This player cooperates with probability 1, since they consider everyone to be good. Player 1 will increment the reputation score of the donor unless the social norm applied is L_4, L_6, L_7, L_8 and the receiver is considered to be bad by player 1.

$$f^i(G,k,l;G,k-1,l+1) = \begin{cases} \frac{k}{N} & \text{if } i \in \{1,3,4,7\} \\ \frac{k}{N}\frac{(k+l)}{N-1} & \text{if } i \in \{2,5,7,8\}. \end{cases} \tag{13}$$

Transition $(G,k,l) \to (G,k+1,l-1)$. This case requires that a player $i > 1$ is chosen to be the donor who is considered to have reputation score $r_{1i} = 1$ by player 1. This player cooperates with probability 1, since they consider everyone to be good. Player 1 will decrement the reputation score of the donor only if the social norm applied is L_2, L_5, L_6, L_8 and the receiver is considered to be bad by player 1.

$$f^i(G,k,l;G,k+1,l-1) = \begin{cases} 0 & \text{if } i \in \{1,3,4,7\} \\ \frac{l}{N}\frac{N-(k+l)-1}{N-1} & \text{if } i \in \{2,5,6,8\}. \end{cases} \tag{14}$$

Transition $(B,k,l) \to (B,k-1,l+1)$. This case requires that a player $i > 1$ is chosen to be the donor who is considered to have reputation score $r_{1i} = 0$ by player 1. This player then has to cooperate, which means that player 1 cannot be the receiver. Player 1 will increment the reputation score of

the donor unless the social norm applied is L_4, L_6, L_7, L_8 and the receiver is considered to be bad by player 1.

$$f^i(B,k,l;B,k-1,l+1) = \begin{cases} \frac{k}{N}\frac{N-2}{N-1} & \text{if } i \in \{1,3,4,7\} \\ \frac{k}{N}\frac{(k+l)-1}{N-1} & \text{if } i \in \{2,5,6,8\}. \end{cases} \tag{15}$$

<u>*Transition* $(B,k,l) \to (B,k+1,l-1)$.</u> This case requires that a player $i > 1$ is chosen to be the donor who is considered to have reputation score $r_{1i} = 1$ by player 1. The receiver then has to be either player 1, against who the donor will defect, or someone player 1 assigns a bad reputation to in case the applied norm is L_2, L_5, L_6, L_8.

$$f^i(B,k,l;B,k+1,l-1) = \begin{cases} \frac{l}{N}\frac{1}{N-1} & \text{if } i \in \{1,3,4,7\} \\ \frac{l}{N}\frac{N-(k+l)}{N-1} & \text{if } i \in \{2,5,6,8\}. \end{cases} \tag{16}$$

<u>*Transition* $(0,k,l) \to (1,k,l)$.</u> This case requires player 1 to be the donor, and that player 1 cooperates with their co-player. The probability is

$$f^i(0,k,l;\ 1,k,l) = \frac{1}{N}\frac{(k+l)}{N-1}. \tag{17}$$

It is equal to $f^i(-1,k,l;\ 0,k,l)$.

<u>*Transition* $(1,k,l) \to (0,k,l)$.</u> This case requires player 1 to be the donor, and that player 1 defects against their co-player. The probability is

$$f^i(1,k,l;\ 0,k,l) = \frac{1}{N}\frac{N-(k+l)-1}{N-1}. \tag{18}$$

It is equal to $f^i(0,k,l;\ -1,k,l)$ in (9).

All other transitions from (s,k,l) to (s',k',l') have transition probability $f^i(s,k,l;s',k',l') = 0$.

We observe that for this reduced Markov chain, the set of recovery states $\mathcal{A}$ is absorbing. As can be verified by looking at the corresponding transition probabilities, leaving this set is impossible: for all i $f^i(G,k,l;G,k',l') = 0$ for $k+l = N-1$ and $k'+l' = N-2$, as well as $f^i(G,k,l;B,k',l') = 0$ for $k+l = k'+l' = N-1$. However, in the cases where one of the four social norms L_4, L_6, L_7, L_8 is employed by the population, there is another absorbing state, namely $(-1,0,0)$ - a full segregation state where player 1 considers everyone else as bad, whereas the remaining players consider player 1 to be bad. Note that $(G,0,0)$ is never an absorbing state.

In the following we will show that for all L_i, both the recovery probability and the expected time to recovery are bounded from above by the corresponding quantitites for the binary reputation case. In analogy to the analysis of the dynamics when reputations are binary, we first visualize the Markov chains M_i (now with quantitative assessment) for the four different cases: $\{L1,L3\}, \{L2,L5\}, \{L4,L7\}, \{L6,L8\}$

(**Supplementary Figure 2– Supplementary Figure 5**). We can then make use of a simple coupling argument to show that these chains will perform better or equal compared to the binary assessment scenario.

We visualize the Markov chains with quantitative assessment by first noting that the transitions of type $(s,k,l) \to (s,k \pm 1, l \mp 1)$ do not change the overall reputations, i.e. the labels "good" and "bad" of any players. We can identify them with internal transitions inside the $3N$ "aggregated" states of type $(s, k+l = t)$ (**Supplementary Figure 1a**), where t is the overall number of players that player 1 considers to be "good". In our illustrations of the chains, we will omit these internal transitions for ease of visualization (**Supplementary Figure 1b**). We note however that some of the remaining state transitions that change the value of s or t are in fact dependent on the value of k, the number of players that player 1 considers to have reputation score $k = 0$. This will become crucial when we compare our model with the case of binary assessment.

First, we consider the case of L_1 and L_3 (**Supplementary Figure 2a**), which have found to be most robust already in the binary case. Both have $\mathcal{A}$ as their only set of absorbing states, and thus a probability of $\rho_1 = 1$ to recover from a single disagreement. When we consider the average steps to absorption, τ_1, we find that the lower bound is unchanged from the binary case: it assumes that we start in state $(1, 0, N-2)$. The expected time until a transition is taken that is not a self loop within the "aggregated" state is $\frac{1}{\frac{1}{N}+\frac{1}{N(N-1)}} = N - 1 + 1 = N - 1$. For the upper bound, we can use a coupling argument. We consider a simplified Markov chain M_1' where we replace the transition probabilities depending on k with their upper bounds obtained by $k = t, l = 0$, and where we erase the states with $s = 0$. As is straightforward to check, this corresponds to the original chain of binary assessment dynamics (**Supplementary Figure 2a**). Intuitively, our argument works by considering that all "bad" moves (i.e., moving downwards or right in the chain) in the quantitative case always have smaller or equal probabilities than the corresponding "bad" moves in the binary case.

More specifically, consider an arbitrary trace T in M_1. If T never takes a transition that changes the value of s, we can associate the identical trace T' in M_1' that never leaves the level $s = G$, since the levels $s = 0$ and $s = 1$ in M_1 are indistinguishable in this case. Otherwise, there is a moment where T has a transition into a state with $s' = s + 1$ or $s' = s - 1$. In these cases, depending on whether T is in $s = 0$ or $s = 1$, the two traces either both take a step into the state where player 1 is considered to be bad, or T remains in a state where player 1 is considered good, whereas he is considered bad in the state that T' reaches (i.e., the middle layer ($s = 0$) of M_1 can act as a buffer). In both cases, we can couple the traces such that T is never below or to the right of T'. The latter holds due to how the "lateral" transition probabilities in the bottom layer ($s = -1$ for M_1 and $s = B$ for M_1') compare. The transition probabilities to the right $f'^1(B, t; B, t-1)$ in the bottom level of M_1' are larger than the corresponding transition probabilities $f^1(-1, k, l; -1, k-1, l)$ in M_1. Additionally, the transition probabilities to the left are the same both in the bottom levels of both chains, as well as in the top level of M_1' and the two top levels in M_1. Finally, the transition probabilities to the left in the bottom level of both chains, $f^1(-1, k, l; -1, k+1, l)$ and $f'^1(B, t; B, t+1)$, are always smaller than those in the upper layer(s)

$f^1(G,k,l;-1,k+1,l)$ and $f^1(G,t;G,t+1)$, respectively. Therefore, it follows that if T' has reached the absorbing state $\mathcal{A}'$ in n steps, T has reached it in n steps with at least the same probability. Thus, we get an upper bound for the number of steps required to reach the absorbing set of states $\mathcal{A}$ in M_1, which is equivalent to the bound calculated for the chain in the binary assessment scenario. Since this bound was found to be $\tau_1' = N+7$, and the lower bound is $\tau = N-1$, we also find that the tight bound of $\tau_1 = \Theta(N)$ holds in the quantitative assessment case.

We can use similar coupling arguments as we look at the remaining cases as well. We proceed with the case of L_2 and L_5 (**Supplementary Figure 3a**), which differs from the previous chain in the positive probability to make a step to the right in an upper level of the chain ($f^2(G,k,l;G,k-1,l) > 0$). Here, the recovery probability is again $\rho_2 = 1$. For the upper bound, we can again look at the Markov chain M_2', which is equivalent to the chain of binary assessment for L_2 and L_5. Following the same arguments as before, we can see that the upper bound on the recovery time again corresponds to the upper bound of the recovery time in the binary case (**Supplementary Figure 3b**), with $\tau_2 \leq \tau_2'$ and τ_2' of order $\Theta(N \log N)$. If we again consider two arbitrary traces T and T', T' can not be in a state with a higher value of s than T (it cannot be "above" T) given that one step downwards is always more detrimental in M_2'. Also, T' cannot be left to T, as the probabilities to move right (i.e. away from the absorbing state) are larger in M_2' than in M_2, and are additionally smaller in the upper level(s) of the chain than in the lower level.

For the remaining cases of $\{L_4, L_7\}$ (**Supplementary Figure 4**) and $\{L_6, L_8\}$ (**Supplementary Figure 5**), we note that we have a second absorbing state that corresponds to a full segregation state: $(-1,0,0)$. In this state, all other players regard player 1 as bad, whereas player 1 themselves regards all other players as bad. Thus, there is a positive probability of not reaching the set $\mathcal{A}$. However, since the binary assessment chains M_4' and M_6' again feature higher probabilities of "bad" moves as defined above, we can still use the same coupling argument as in the two cases before. Both the recovery probability and expected time to recovery of the quantitative chain M_4 (**Supplementary Figure 4**) are bounded by the corresponding properties in M_4' by way of $\rho_4 \geq \rho_4' \geq 1 - 2/(N-1)!$ and $\tau_4 \leq \tau_4' \leq 2(N-1) \cdot (e-1) + o(1)$. The lower bound for τ_4 remains the same as in the binary case and is identical to the lower bound in the first case, with $\tau_4 \leq N-1$, such that we again get $\tau_4 = \Theta(N)$.

The same reasoning holds for M_6 (**Supplementary Figure 5**), which differs from M_4 again in the positive probability to make a step to the right in an upper level of the chain ($f^6(G,k,l;G,k-1,l) > 0$). We get, by comparing with the bounds for the binary case, that $\rho_6 \geq 1 - \frac{1}{N}$ and $\tau_6 \leq N \cdot H_N - N$, with H_N the N-th harmonic number $\sum_{n=1}^{N} \frac{1}{n}$.

These are very rough upper bounds. In fact, when we take a look at the actual recovery times of the system, we find that in all eight cases, $\tau = O(n)$, i.e. that recovery time is approximately linear for all leading eight norms. We show the resulting plot in **Figure S3a**. When we do linear regression on these curves, $\tau_i \approx N$ for $i \in \{1,3,4,7\}$ and $\tau_i \approx 1.3N$ for $i \in \{2,5,6,8\}$. This is a substantial improvement over the recovery times for the case of binary reputations. We additionally find that the expected number of defections until recovery goes towards zero as the population becomes large: a single perception error

typically triggers no further defection (**Figure S3b**). We note that these results are obtained when the population starts in the state $(0, N-2, 0)$, i.e. the state where all entries of the starting image matrix are zero except for one negative entry. This corresponds to the starting state of our simulations in the main text. Alternatively, if we let our system start in the state $(1, 0, N-2)$, recovery occurs at $\tau \approx N$ for all leading eight strategies, including L_2, L_5, L_6, L_8.

Supplementary Note 2: Characterization of successful strategies

In analogy to the work of Ohtsuki and Iwasa[2], we now explain the characteristics of those third-order strategies that are successful both under public information as well as private and noisy information. For this axiomatic approach, we now assume that players use quantitative assessment, since binary assessment does not lead to the evolution of cooperation once information is not public.

In the following, we use notation similar to previous work, adapted to our model of quantitative assessment. We again distinguish between reputation scores $r_{ij} \in [-R, R]$, and the corresponding overall judgments (labels) as "good" or "bad", which arise from comparing these scores with the threshold S. The assessment (i.e. adding or subtracting from the score) of an action X by a donor with label A towards a recipient with label B according to the social norm is denoted by $d(AB, X) \in \{-1, +1\}$. The action (i.e. to cooperate or to defect) prescribed by the social norm for a donor with overall label A and a recipient with label B is denoted as $p(AB) \in \{C, D\}$.

In the public information scenario, Ohtsuki and Iwasa identified the following four properties that a third order strategy needs to fulfill to be successful in letting cooperation evolve.

1. Maintenance of cooperation. Assuming that a high reputation leads to a benefit that is higher than the cost of help, most players should cooperate with each other for a norm to be successful. This requires

$$p(GG) = C \ \text{ and } \ d(GG, C) = +1 \tag{19}$$

2. Identification of defectors. Players using the social norm need to be able to identify defectors. An $ALLD$ player should not get the chance to improve their reputation, and should instead be labeled as "bad" as soon as possible. Thus, the following condition that decreases the reputation score of a player who defects against a good opponent must hold:

$$d(GG, D) = -1 \ \text{ and } \ d(BG, D) = -1 \tag{20}$$

3. Justified punishment. A player who defects against an opponent judged as bad should refuse cooperation, and not be punished for it themselves. This means

$$p(GB) = D \ \text{ and } \ d(GB, D) = +1 \tag{21}$$

4. Apology and forgiveness. A player who erroneously defected against an opponent should be able

to regain their lost reputation once they demonstrate their goodwill by cooperating with a good opponent. This gives

$$p(BG) = C \text{ and } d(BG, C) = +1 \quad (22)$$

We note that these four required properties are independent of whether players use binary or quantitative assessment. These conditions fix five elements (bits) of a successful norm's assessment rule. In Ohtsuki and Iwasa's original work, three bits were then left unspecified, giving the leading eight. However, if we consider the setting where information is private and noisy, we need to specify one more bit with the following condition:

5. Suspicion. To be successful under private and noisy information, norms need to be less gullible than in the case of public information, and need to be more suspicious of known defectors. In particular, they cannot allow defectors to gain an improved reputation score when they defect against another defector, since this would allow $ALLD$ to invade. Rather, repeated defectors should continously lose reputation. This requires

$$d(BB, D) = -1 \quad (23)$$

With these requirements, four of the leading eight norms remain: L_1, L_2, L_7, L_8. They are exactly the four norms that we see being able to evolve under private and noisy information, as long as they use quantitative assessment. The norms L_3, L_4, L_5, L_6 in contrast are more gullible, and let defectors regain some of their reputation by defecting against another of their kind.

We note however that among the successful norms, L_8 has the fewest opportunities for a player labeled as bad to improve his score and be labeled good (**Fig. 1a**). For example, an unconditional cooperator easily gets a bad label in the eyes of an L_8 player. This explains why we see the lowest abundance and cooperation rate in equilibrium in L_8 out of all four successful norms, and why the success of L_8 is also more sensitive to an increased number of reputation ranks (**Fig. 5**).

Supplementary References

[1] Hilbe C, Schmid L, Tkadlec J, Chatterjee K, Nowak MA. Indirect reciprocity with private, noisy, and incomplete information. Proceedings of the National Academy of Sciences USA. 2018;115:12241–12246.

[2] Ohtsuki H, Iwasa Y. The leading eight: Social norms that can maintain cooperation by indirect reciprocity. Journal of Theoretical Biology. 2006;239:435–444.

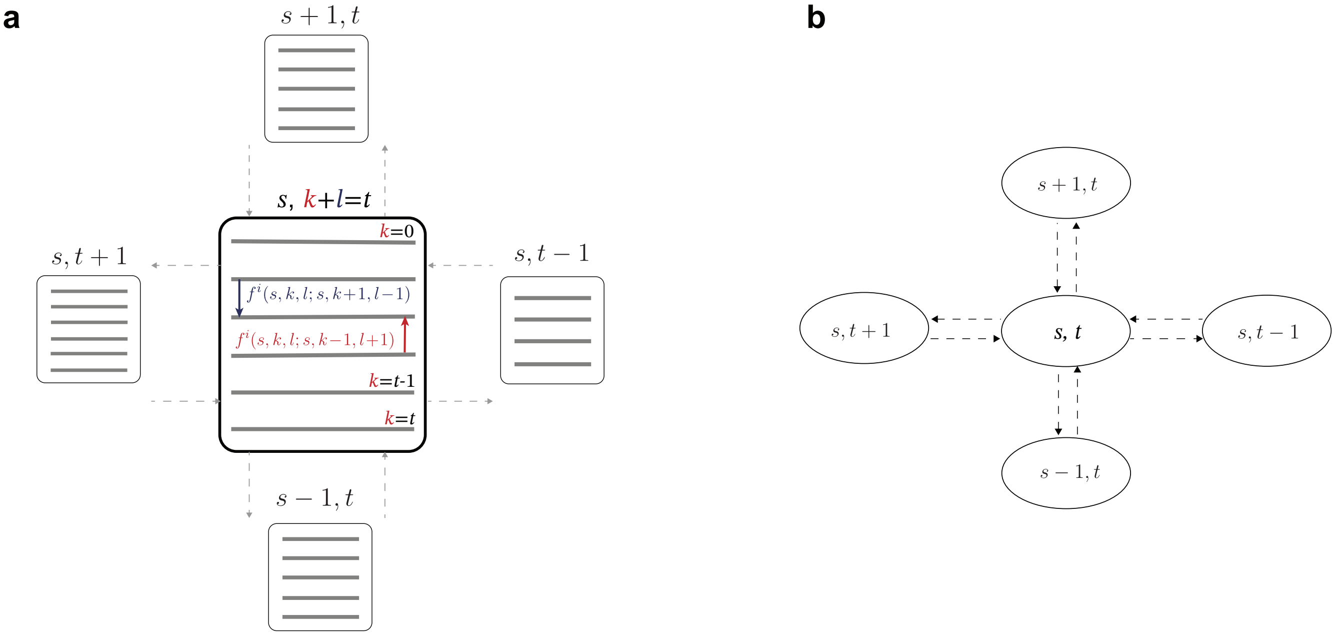

Supplementary Figure 1: The states of the reduced Markov chains modeling assessment dynamics. **a,** Aggregated states of the type $(s, k+l)$, with s the assessment of player 1 in the eyes of the other players, and $k+l$ the number of players that player 1 assesses as good. They aggregate states (s, k', l') with $k'+l' = k+l$), with internal ("hidden") transitions that change the value of k and l while keeping their sum constant. **b,** For ease of visualization, we only show the aggregated states and omit the internal states when we illustrate the Markov chains in the following. Note however that the internal state can determine the transitions out of a state.

a

b

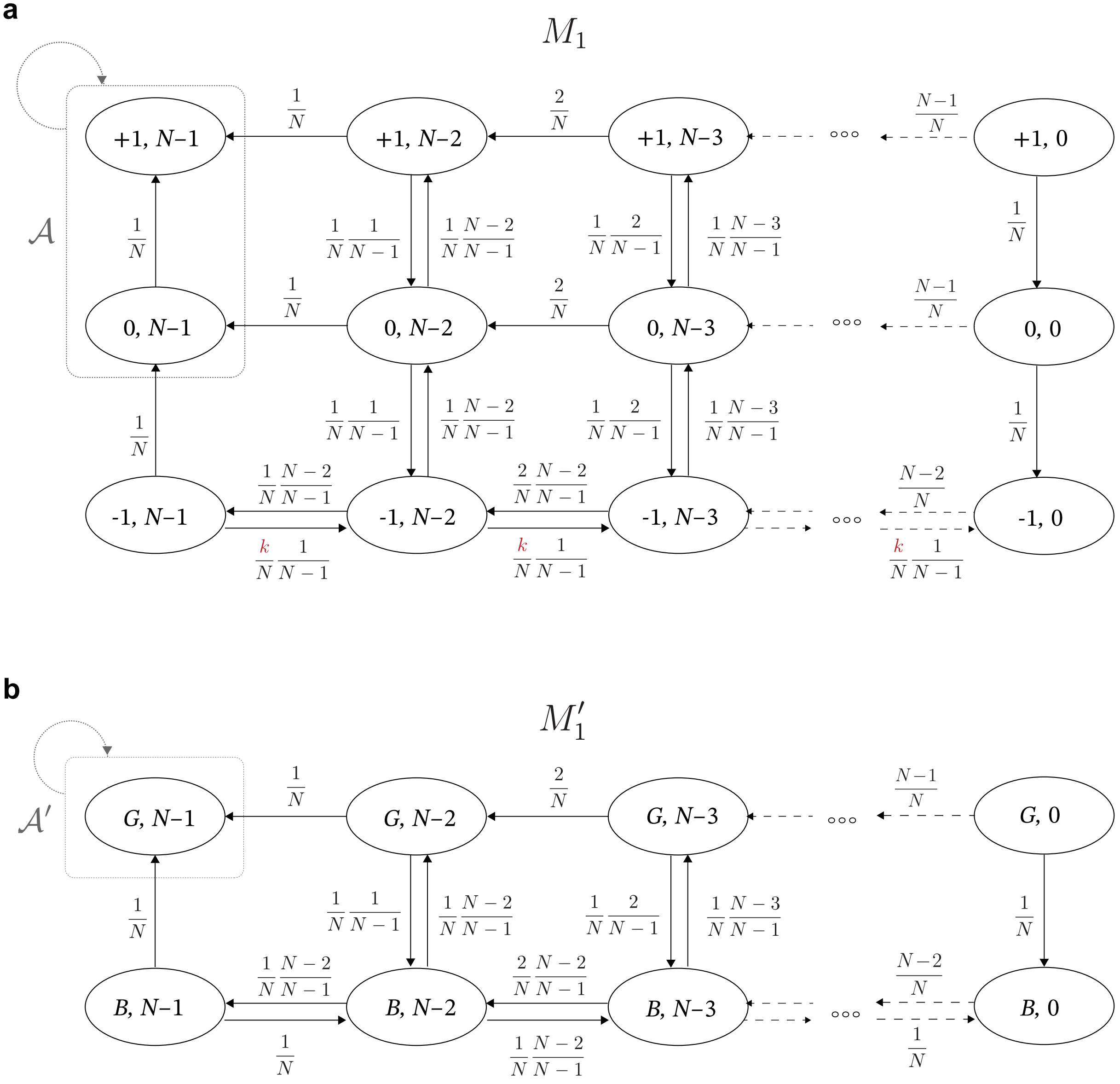

Supplementary Figure 2: Markov chain modeling the dynamics of L_1 and $L3$ for recovery. a, The full Markov chain M_1 has three "levels" corresponding to the three different values that s can take. **b,** For the upper bound, we consider a chain where states with $s = 0$ are erased and transition probabilities to the right that are proportional to k are upper bounded by $k + l$. This is equivalent to the chain for the binary assessment case, M_1'.

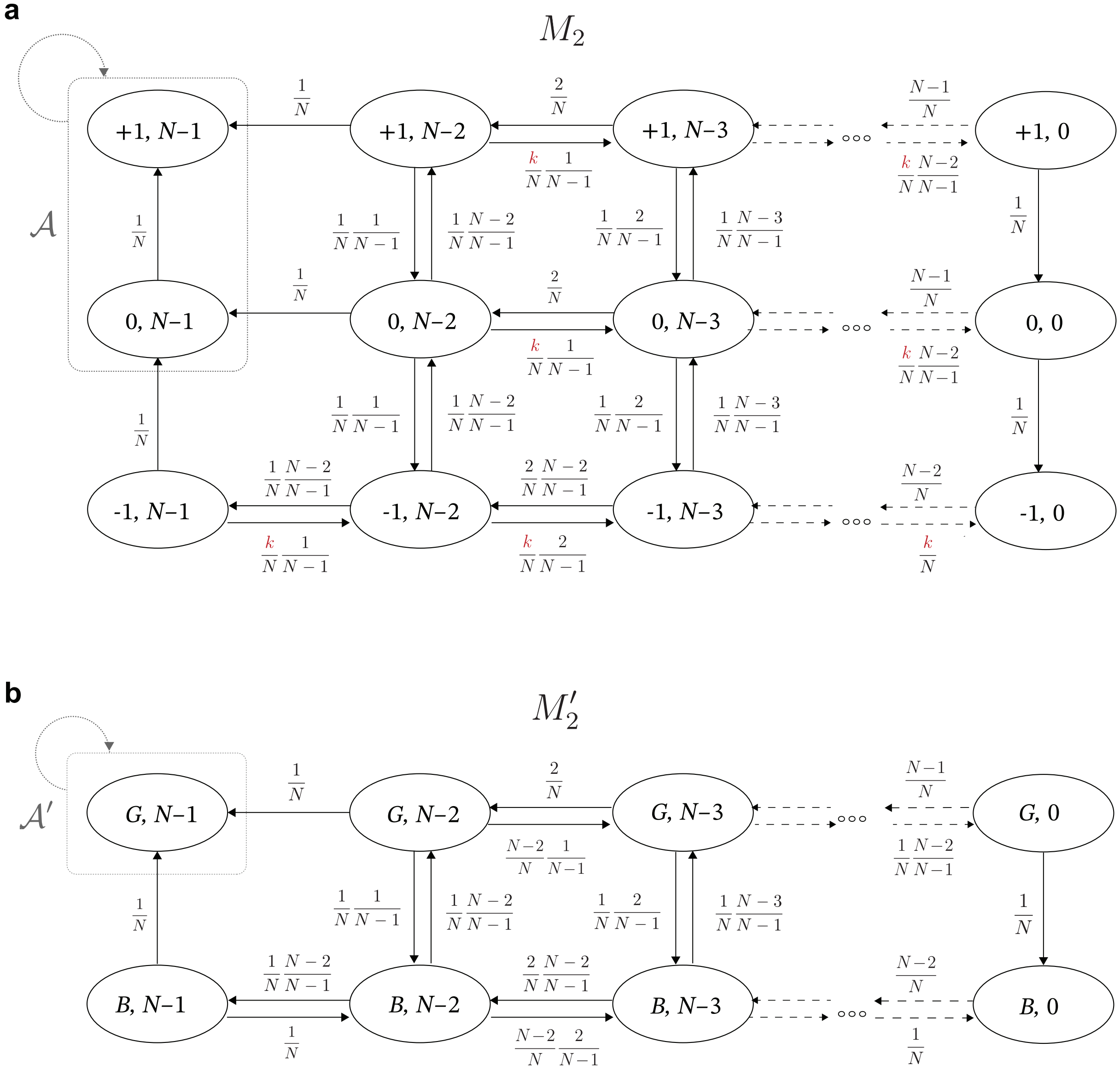

Supplementary Figure 3: Markov chain modeling the dynamics of L_2 and L_5 for recovery. a, The full Markov chain M_2 has three "levels" corresponding to the three different values that s can take. **b,** For the upper bound, we again consider a chain where states with $s = 0$ are erased and transition probabilities to the right that are proportional to k are upper bounded by $k + l$. This is equivalent to the chain for the binary assessment case, M_2'.

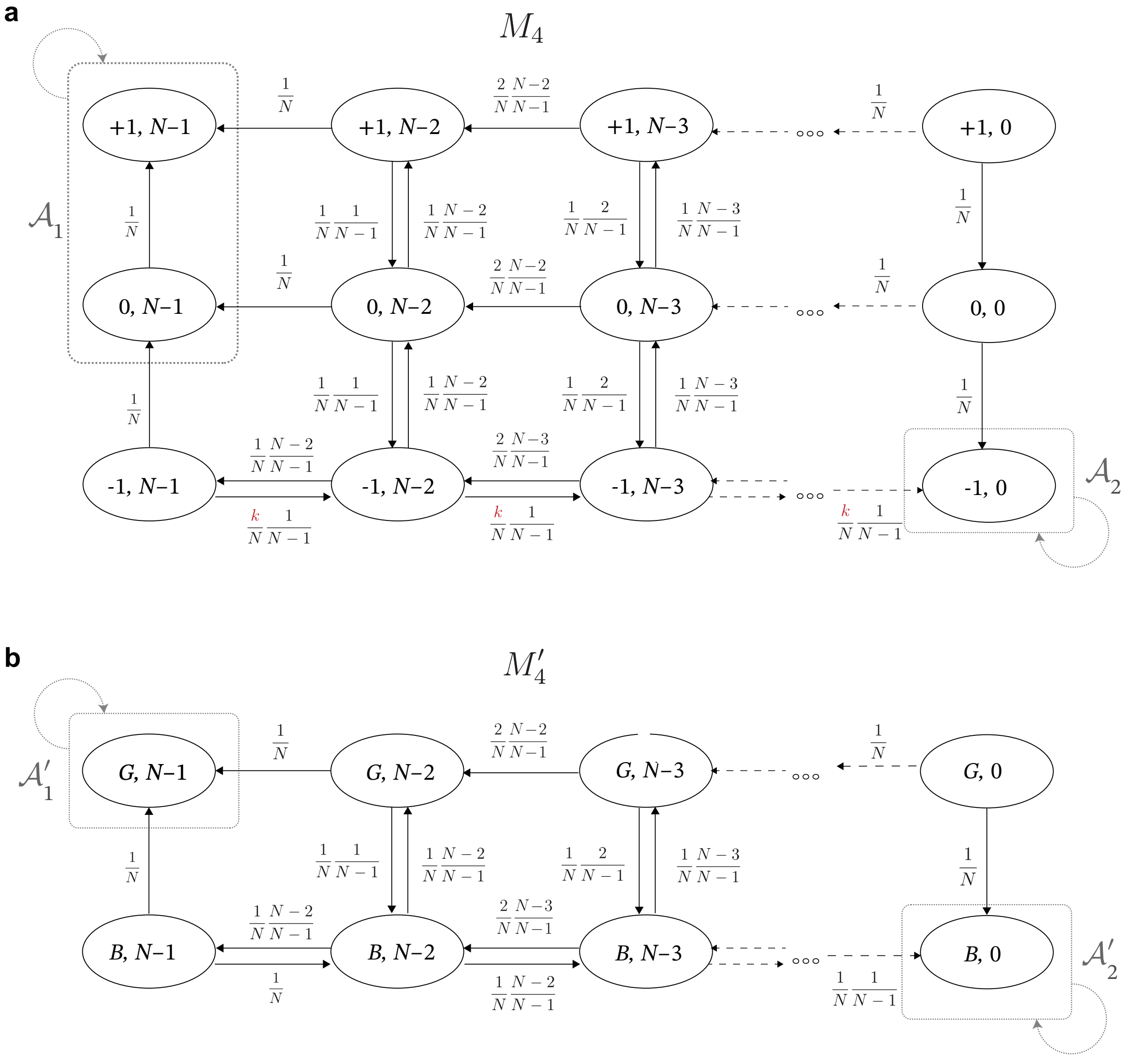

Supplementary Figure 4: Markov chain modeling the dynamics of L_4 and L_7 for recovery. a, The full Markov chain M_4 has three "levels" corresponding to the three different values that s can take. **b,** For the upper bound, we consider a chain where states with $s = 0$ are erased and transition probabilities to the right that are proportional to k are upper bounded by $k + l$. This is equivalent to the chain for the binary assessment case, M_4'.

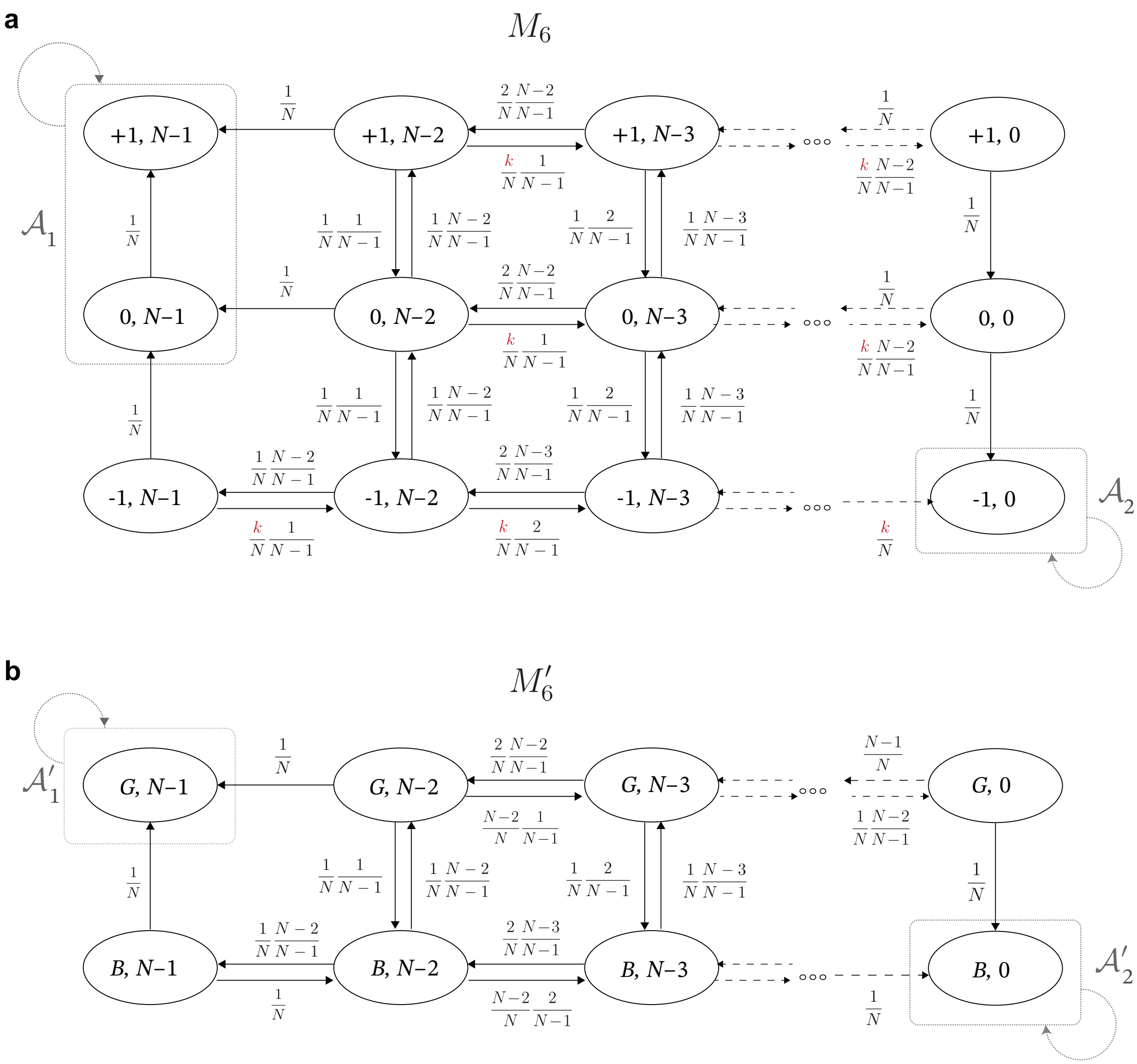

Supplementary Figure 5: Markov chain modeling the dynamics of L_6 and L_8 for recovery. a, The full Markov chain M_6 has three "levels" corresponding to the three different values that s can take. **b,** For the upper bound, we consider a chain where states with $s = 0$ are erased and transition probabilities to the right that are proportional to k are upper bounded by $k + l$. This is equivalent to the chain for the binary assessment case, M'_6.
